# Addressing mental health deserts: a geographic and economic analysis of mental health service gaps in Houston

**DOI:** 10.3389/fpubh.2025.1571183

**Published:** 2025-10-16

**Authors:** Damien Kelly, Chakema Carmack

**Affiliations:** ^1^HEALTH Research Institute Center for Addictions Research and Cancer Prevention – RCMI, Community Engagement Core, University of Houston, Houston, TX, United States; ^2^Department of Psychological, Health, and Learning Sciences, University of Houston, Houston, TX, United States

**Keywords:** mental health deserts, distressed community index (DCI), access to mental health services, mental health policy, economic disparities in health care

## Abstract

**Introduction:**

The increasing prevalence of mental health challenges in the United States, particularly among low-income communities, has highlighted significant disparities in access to mental health services. This study investigated the concept of “mental health deserts,” areas with insufficient mental health care providers, in the city of Houston, Texas. By utilizing the Distressed Community Index (DCI) to identify economically disadvantaged areas and sampling Psychology Today’s database of over 3,000 licensed therapists in the Houston area to map mental health service availability, this research aimed to determine the existence and extent of mental health deserts in Houston.

**Methods:**

The study employed a food desert model as a conceptual framework, comparing the availability of mental health services in economically deprived areas. Analysis of Variance (ANOVA) was used to assess the relationship between DCI scores and the number of mental health professionals, types of mental health service professionals, and the education level of providers among *N* = 96 zip codes and 395 therapists in the city of Houston.

**Results:**

Results showed that certain low-income areas in Houston, particularly those with higher DCI scores (i.e., more distressed), lack adequate mental health resources, possibly qualifying them as mental health deserts.

**Discussion:**

These areas demonstrate a significant need for targeted interventions at multiple levels to improve access to mental health care. Implications to inform policymakers and healthcare providers about the critical need for mental health infrastructure in economically distressed communities are discussed. By identifying and addressing these gaps, this research aims to contribute to the broader effort of reducing mental health disparities and promoting wellness among underserved populations. The present study is a preliminary examination and meant to initiate more research on mental health deserts in areas with significant need for awareness, intervention, and policy attention.

## Introduction

Mental health is critical to well-being, and like physical health, it factors in an individual’s ability to function as a productive citizen and community member. Mental health impacts many aspects of our daily lives, such as productivity at work and interactions with friends and family (1). The importance of mental health has become an ever-pressing issue in the United States with the rising number of adults living with mental health challenges (2). Recent estimates have shown that 21% of adults in the United States are experiencing a mental illness, which translates to over 50 million Americans (3). Of those Americans who reported experiencing mental health needs, 28% reported they were unable to receive proper mental health treatment. Most reported they could not receive care due to the cost (3).

According to the Substance Abuse and Mental Health Services Administration (SAMHSA), results from a 2019 National Survey on Drug Use and Health reported mental health issues among women (28%) and men (15%), with white adults more likely to report having a mental health issue as opposed to persons of color (SAMHSA, 2020). However, according to Budhwani et al. (4), despite having lower reported rates of mental illness, individuals from communities of color may experience more persistent mental health challenges. They are less likely to pursue or receive adequate treatment, which can lead to prolonged or untreated conditions over time and health disparities in population health (5).

Likewise, the demand for mental health services is rising along with the cost. Cantor et al. (6) found that utilization and spending rates for mental health care services among commercially insured adults increased by 53.7% in 2022. However, are those services available to adults living in low-income areas? Families with lower incomes have been linked to negative mental health outcomes, substandard health outcomes, and increased risk for mental health instability, including higher rates of child mental health concerns [Hodgkinson et al., 2017; (7)]. Socioeconomic inequality contributes to healthcare disparities and can lead to increased healthcare expenses (8). These problems can persist across an individual’s lifespan. Despite the growing mental health needs of families of lower socioeconomic status, few gain access to high-quality mental health services. Factors such as elevated crime rates, food deserts, and limited upward mobility intensify stress in these communities. Although government-funded safety nets may offer some assistance in this area, families utilizing government-sponsored safety net programs such as Temporary Assistance for Needy Families (TANF), Supplemental Nutrition Assistance Program (SNAP), and Supplemental Security Income (SSI) benefit less compared to other industrialized countries (7).

Although economic barriers to mental health persist in low-income families, individuals can experience additional barriers to seeking mental health care as well. Those living with mental health challenges might experience prejudice in seeking mental health (2). In various communities, social taboos remain about mental health. Lack of mental health-seeking behavior can also lead to self-medication with drugs and alcohol (2, 9). Research indicates there is a strong connection between homelessness and mental health, highlighting how factors such as medical mistrust, discrimination, poor health literacy, and victimization exacerbate the challenges faced by individuals in these populations. Fornaro et al. (10) emphasized that individuals experiencing homelessness are particularly vulnerable to a variety of health- related issues, such as substance use disorders, infectious diseases, and mental illnesses, including depression and schizophrenia. Barry et al. (11) reported that 67% of individuals experiencing homelessness suffer from mental health illnesses. The researchers noted that individuals experiencing homelessness may distrust medical professionals due to receiving inadequate healthcare and experiencing discrimination within medical systems. These underlying systemic issues may lead to patients’ overall suboptimal care. Moreover, limited health literacy compounds these challenges, making it harder for homeless and minority populations to navigate healthcare systems or fully understand their treatment options.

Additionally, exposure to disasters has been related to various mental health outcomes that can impact individuals, specifically those with lower socioeconomic status (1). Zürcher et al. (12) found that during and after stressful events, such as the COVID pandemic, individuals may initiate or exacerbate systems of anxiety, depression, and post-traumatic stress disorders. Indeed, the pandemic led to increased anxiety, depression, and stress in lower-income families (Ling, 2021), and thus, substantial challenges remain, particularly for low-income communities. Barriers include limited internet access, inadequate connectivity, and potential distractions that may require the client’s attention [Uhlmann et al., 2021; (13)]. These socially deterministic and psychosocial barriers faced by disadvantaged communities are also important considerations for mental health and show a need for community population mental health.

## Conceptual framework

We proposed that mental health deserts may follow a similar conceptualization to food deserts. Here, we briefly outline the features of food deserts and then offer a similar model for the investigation of mental health deserts. The concept of a “food desert” has been a focal point in public health and urban planning research since 1995 (14). A food desert is commonly defined as a region lacking adequate access to vendors offering nutritionally dense foods, a condition often observed in low socioeconomic areas (13, 15). Over time, awareness of the term has extended beyond the scientific community, raising public understanding of these regions and how they are identified.

**Figure 1:**
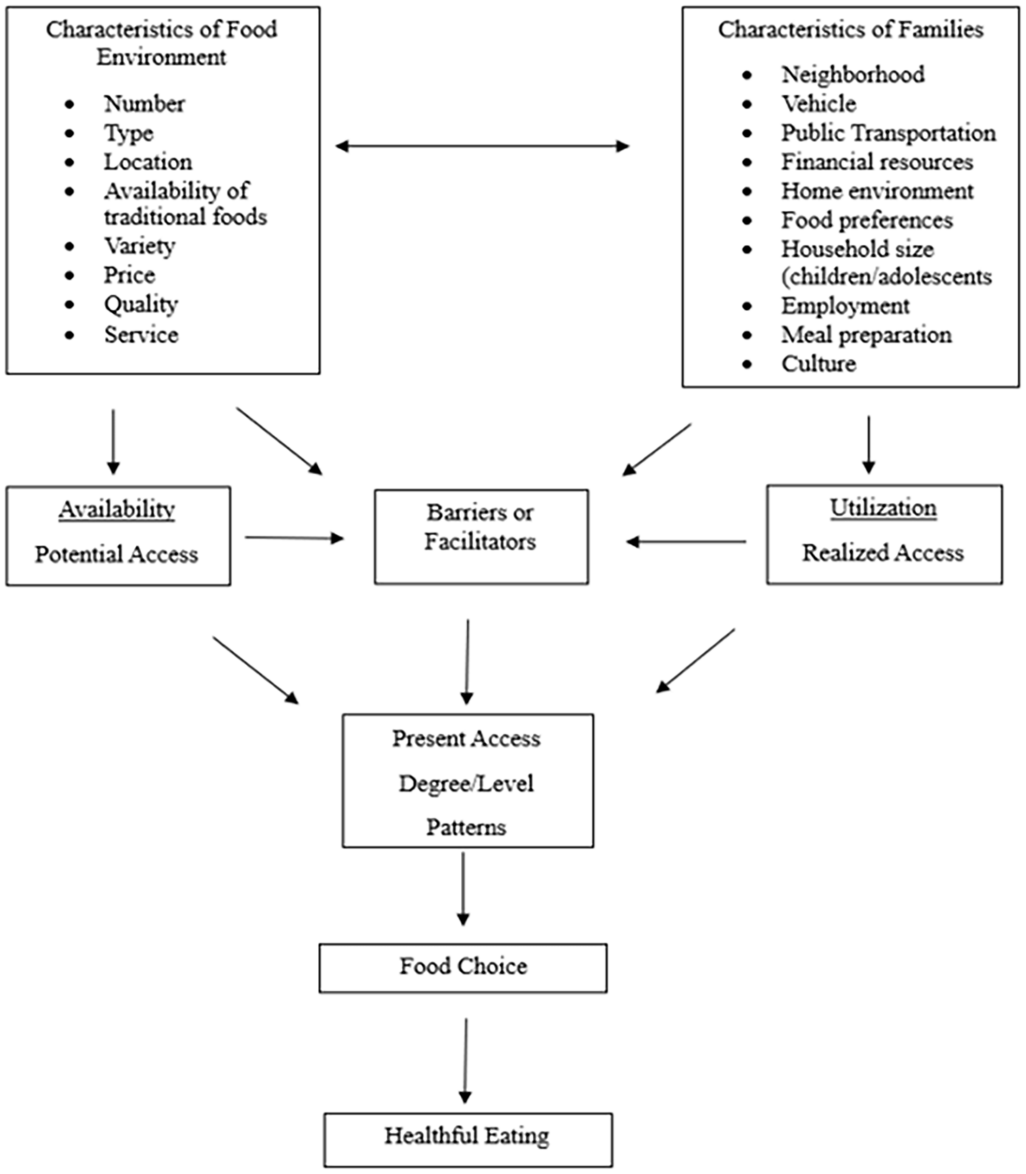
Conceptual model of food access (16).

Sharkey et al. (16) explored access to food stores in rural Texas neighborhoods, focusing on traditional outlets, such as supermarkets and grocery stores, and non-traditional ones, including dollar stores and mass merchandisers. Their research examined the availability of fresh fruits and vegetables by integrating food store data with U. S. Census information. The study also investigated the role of neighborhood factors, such as socioeconomic status and automobile ownership, in shaping levels of food access. Figure 1 illustrates the conceptual framework for food access as developed by Sharkey et al. (16). The existence of food deserts in low-income urban areas has several detrimental effects on community health and well-being (17, 18). Underserved areas face limited access to nutritious food, as large grocery chains are unwilling to invest in these neighborhoods due to perceived economic risks. This results in individuals residing in these areas having fewer food choices and relying on lower-quality or less nutritious options, such as fast food and processed goods. Previous research has indicated that the availability of fresh meats, fruits, and vegetables is particularly restricted, and even when available, these healthy options tend to be more expensive, making them less accessible to those living on limited incomes [DePaiva et al., 2023; (17, 19)]. Crowe et al. (20) explored the consequences of food deserts and the related social stressors that can exacerbate health issues in these communities. Individuals living in food deserts spend a significant portion of their income on transportation to distant grocery stores. This can be particularly burdensome for those without a vehicle or access to reliable public transportation. This lack of convenience not only increases the cost of obtaining food, but also leads to more stress and reduced overall quality of life. Taken together, previous research has indicated that a lack of access to affordable, healthy food (i.e., the socio-political and built environment) is associated with poor dietary habits, contributing to the rise of diet-related diseases such as obesity, diabetes, and cardiovascular issues. The combination of food insecurity and socio-economic pressures creates a cycle of stress that negatively affects both physical and mental health, leading to a greater prevalence of conditions like anxiety and depression.

Although ameliorating food deserts may have other desirable results, such as job creation and economic stimulation, from a behavioral perspective, the consumer (of either fresh foods or therapy) is the end-user whose health (physical or mental) may be improved by its utilization. It is worthwhile to consider that the mental health concerns of disadvantaged communities may be associated with the lack of perceived and actual availability of mental health providers within their community.

## The present study

**Figure 2:**
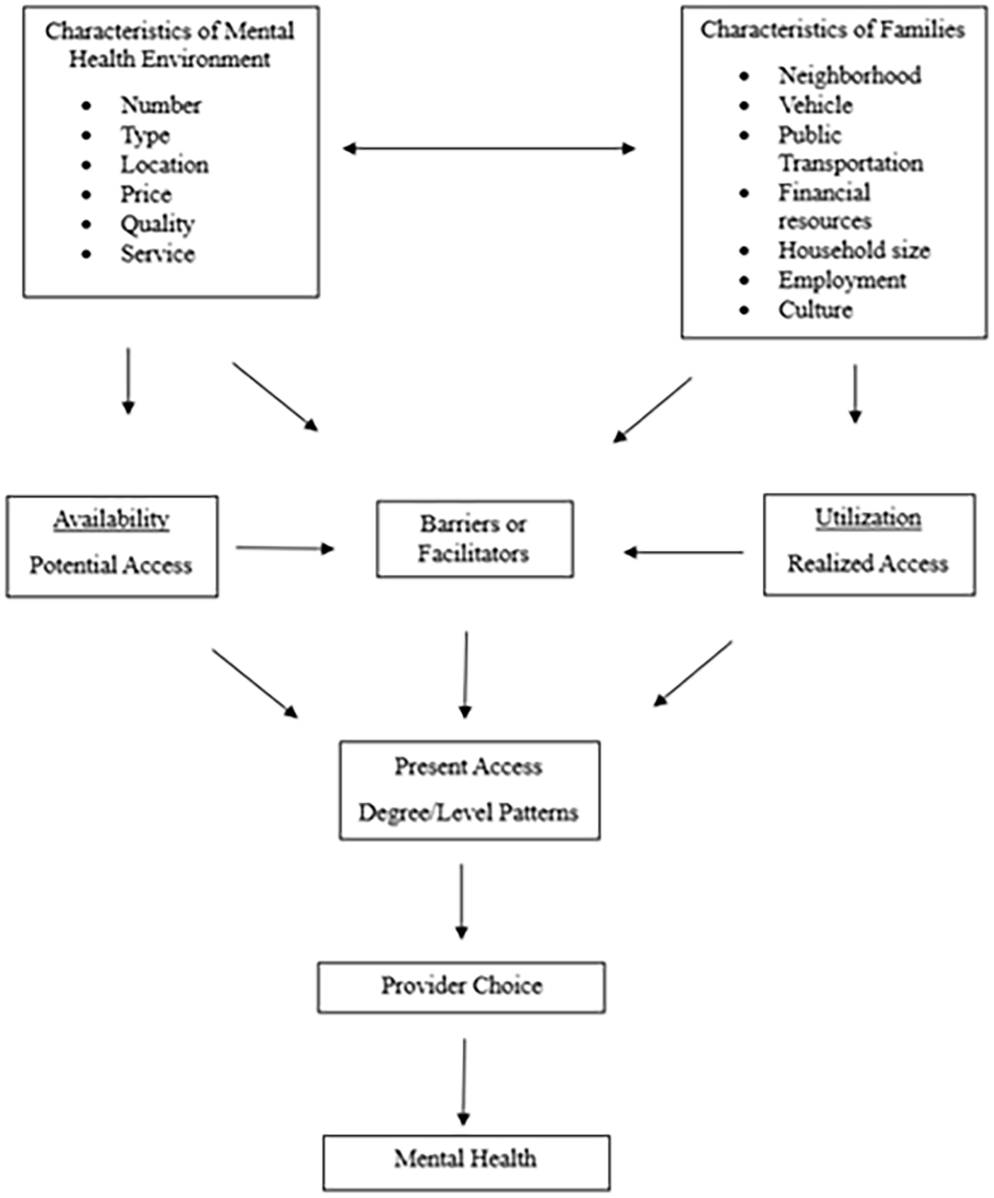
Conceptual model of mental health deserts.

The present study presents an initial consideration for the examination of mental health deserts and presents descriptive and inferential associations of provider availability and neighborhood distress. Based on the previous literature regarding food deserts (Figure 1), Figure 2 shows the proposed conceptualization of mental health deserts. By using a food desert model, we explored socioeconomic advantaged and disadvantaged zip codes in Houston to determine what areas may preliminarily qualify as mental health deserts. We examined the areas of Houston, Texas, and the unavailability of mental professionals and services as barriers to access across communities. To analyze availability and access to mental health professionals, we utilized the Distressed Community Index (DCI) scores for populations within the city of Houston. These distress scores accounted for key factors such as poverty rates, median household income, and demographic composition. By considering these factors, we aimed to identify evidence for the existence of mental health deserts and targeted areas of opportunity to better understand mental health service utilization in socioeconomically distressed communities. The characteristics of the mental health environment were assessed by examining the number of mental health providers within specific zip codes, as well as the providers’ education levels and particular mental health service types. Through this initial investigation, we intended to determine whether a statistically significant disparity exists between the distressed communities and mental health professionals’ availability for their community members.

This study was designed to initiate a public health discourse on the definitions and public health purposefulness of coining *mental health deserts* and show a need (if any) for mental health intervention opportunities in these communities. Economically distressed and at-risk areas of Houston, Texas may qualify as mental health deserts, indicating disproportionate access to area-based mental health care. Both unique and common mental health concerns in distressed communities have been previously established in the literature, implying a need for community mental health. Ultimately, this research will help demonstrate the need for programs and facilities in addressing the mental health needs of distressed communities.

## Methodology

### Study sample

According to the U.S. Postal Service, of the 200 zip codes listed for the Houston Metropolitan Statistical Area, 97 are specifically associated with the city of Houston proper. Zip codes for suburban areas such as Kingwood, Woodlands, Sugarland, Pearland, Katy, and others are part of the broader metropolitan region but not within the city limits of Houston (21). One of the 97 zip codes (77053) was found to contain no statistical information. Thus, the final study sample consisted of *N* = 96 Houston city proper zip codes were used for the present study. Public and private mental health providers were identified utilizing Psychology Today’s database (Psychology Today, 2019) of over 3,000 licensed mental health professionals in the U.S. By using this database, we obtained the list of applicable mental health providers who serve Houston. In total, there were *N* = 395 registered mental health professionals who practiced within Houston city zip codes.

### Measures

#### Distressed community index (DCI)

The DCI is a data tool developed to compare the economic well-being of U. S. communities by zip code (22). The DCI is maintained by the Economic Innovation Group, and the data was collected from the U. S. Census Bureau American Community Survey and the Census Bureau’s Business Patterns survey. DCI is a comprehensive socioeconomic ranking by zip code. DCI is available for all zip codes with more than 500 people, which captures 99% of the American population. The seven factors that comprise the DCI are the unemployment rate, poverty rate, median income ratio, percentage of high school degree (education level), percentage change in business establishments, housing vacancy rates, and percentage change in employment (i.e., job growth) (8, 22, 23). The DCI scores range from 0 to 100, with a higher score indicating more community distress: prosperous (*N* = 7; range 7.9–17.8), comfortable (*N* = 10; range 20.8–37.4), mid-tier (*N* = 18; range 40.6–58.8), at- risk (*N* = 19; range 61.0–78.8), and distressed (*N* = 42; range 80.1–97.0). This study will define Houston area low-income communities based on their distressed community index (DCI) score. Take this out completely. The paper does not include a list of all zip codes.

##### Mental health providers

Psychology Today’s (2019) list of mental health professionals consisted of 23 mental health titles including Ph.D. psychologists, licensed professional counselors, and licensed master’s social workers, among others. We used 15 of the 23 categories and collapsed them into four categories of mental health specialties: licensed professional counselors (LPC), practicing psychologists (PPsyc), mental health social workers (SW), and marriage and family therapists (LMFT). LPCs included licensed professional counselors, associate counselors, counselor supervisors, and licensed chemical dependency counselors.

Practicing psychologists were terminal degree counseling practitioners, including Doctor of Philosophy, Psychology, Social Work, Education, and Behavioral Health (PhD/PsyD/DSW/EDD/DBH). SWs included master social work practitioners and licensed social workers, licensed master social work practitioners, and licensed clinical social workers (MSW/LICSW/LCSW/LMSW). Marriage and family counselors included licensed marriage and family therapists and licensed marriage and family therapist associates (LMFT-A/LMFT). Practitioner titles that were omitted included music and art therapists, practicing students/interns, biodynamic craniosacral therapists, eye movement desensitization therapists, and nurse practitioners. There are approximately 392 mental health professionals registered to serve in Houston, Texas, from August 1, 2023 to July 31, 2024.

### Analysis procedure

The research was conducted to identify mental health service gaps in Houston, Texas. We compiled the data to identify each zip code: DCI, racial/ethnic composition, poverty rate, and a corresponding tabulation of the mental health service providers, type of mental health professional, and online availability for mental health services.

Data was screened for analysis suitability and to ensure the assumptions of the model. Descriptives for racial/ethnic composition, poverty rate, and total number of mental health professionals were calculated for each zip code.

The ANOVA procedure was used to construct six models to identify significant differences based on six independent research inquiries. DCI categories (Distressed, At-risk, Mid-Tier, Comfortable, or Prosperous) served as the independent variable. Dependent variables included: (a) Number of mental health professionals who serve the zip code, (b) Number of online therapists available who serve the zip code, (c) Number of mental health professional specialties who serve the zip code. Model 1 examined significant differences between DCI and the total number of mental health professionals. Model 2 examined significant differences between DCI and the online availability of mental health professionals in the zip code. Models 3–6 examined significant differences between DCI and mental health professional specialties (LCPs, PPsyc, SWs, and MFTs). Each model 1–6 was estimated using a univariate test of between-subjects effects, with the population size for each zip code entered into the model as a covariate, since estimates may be unstable if the zip code population (the denominator) is small. For all models, we hypothesize that there will be significant differences regarding mental health professionals that vary by DCI category using a 0.05 *a priori* alpha level for evaluating the *p*-value. All models were analyzed using the Statistical Package for the Social Sciences (version 25.0).

## Results

Descriptives were analyzed for each zip code and its DCI rating. Table 1 shows the DCI rating, DCI category label, and the average number of mental health professionals in each DCI area. This resulted in *N* = 7 zip codes designated as prosperous; *N* = 10 designated as comfortable; *N* = 18 designated as mid-tier; *N* = 19 designated as at-risk; and *N* = 42 designated as distressed.

**Table 1:** Average number of mental health professionals practicing in Houston zip codes.

DCI	*N*	Mean	S.D.
1 Distressed	42	1.90	6.191
2 At-risk	20	2.20	2.726
3 Mid-tier	17	6.18	6.136
4 Comfortable	10	8.60	8.030
5 Prosperous	7	11.00	10.985
Total	96	4.08	6.885

**Figure 3:**
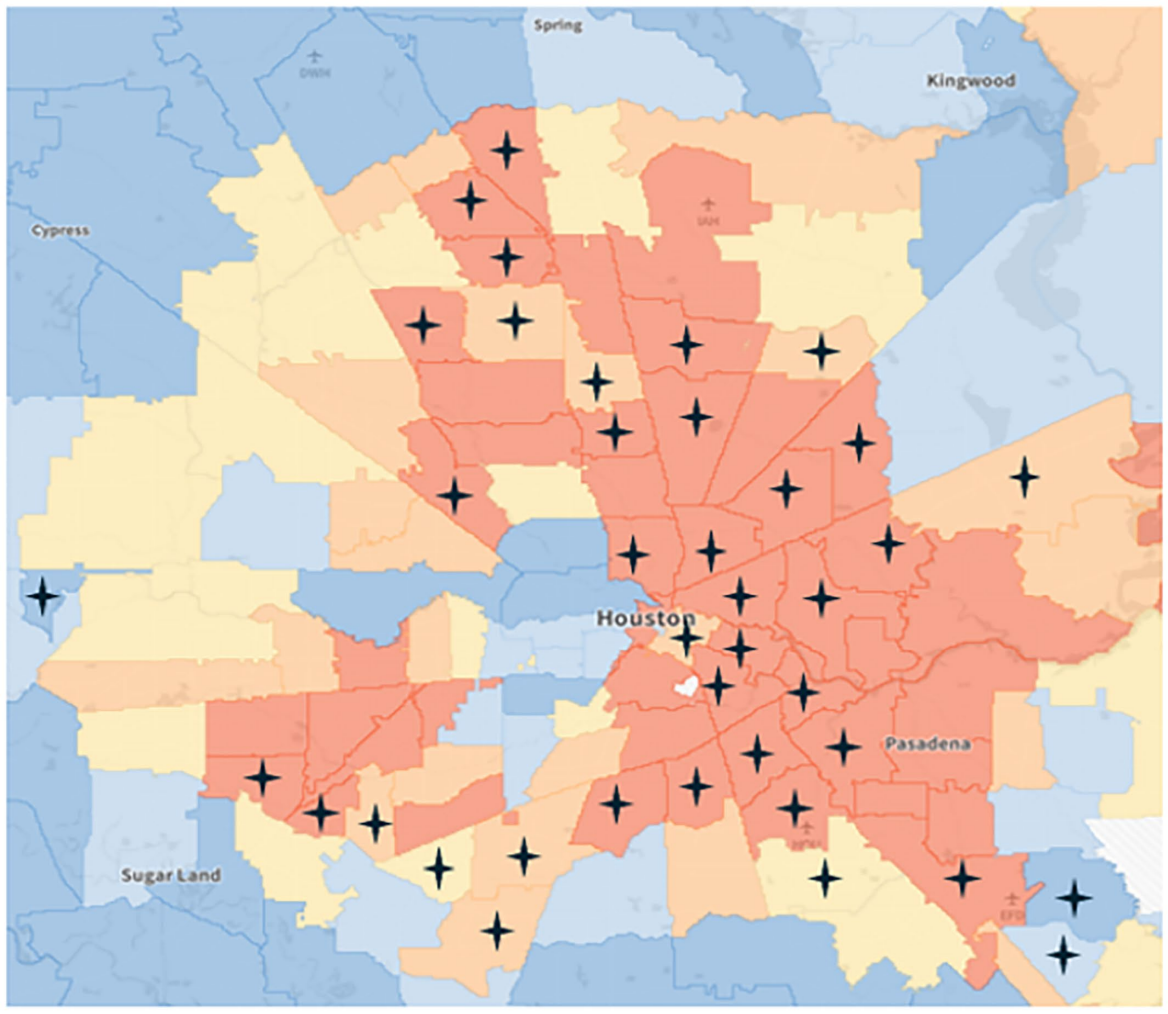
DCI map of Houston: areas of lacking mental health providers.

Descriptive statistics showed that out of the 96 Houston zip codes, *N* = 38 had no mental health practicing professionals. Among these, *N* = 26 were situated in distressed communities, underscoring a pronounced disparity in access to mental health services in areas of higher need. Additionally, *N* = 7 zip codes without professionals were classified as at-risk, followed by *N* = 2 in mid-tier and prosperous communities, and *N* = 1 comfortable community with no practicing professionals. This distribution highlights inequities in the availability of mental health resources across varying levels of community socioeconomic well-being. Figure 3 geographically illustrates the Houston zip codes lacking mental health professionals. Starred areas indicate no mental health professionals registered in the area. The dark orange zip codes indicate a distressed area; light orange zip codes indicate an at-risk area; yellow zip codes indicate mid-tier areas; light blue zip codes indicate a comfortable area; and dark blue zip codes indicate a prosperous area.

Model 1 examined whether there was a significant difference in the level of DCI distress for a particular zip code and the number of mental health professionals, while controlling for zip code population size. Results showed a significant difference between the levels of neighborhood distress and the number of mental health professionals in DCI zip codes of Houston (*F* (4.91) = 5.5, *p* < 0.001). Distressed zip codes (*M* = 1.9 ± 6.2) were significantly different from both comfortable (*M* = 8.6 ± 8.0) and prosperous (*M* = 11.0 ± 11.0) zip codes.

Additionally, at-risk zip codes (*M* = 2.2 ± 2.7) significantly differed from prosperous (*M* = 11 ± 11.0) zip codes regarding the number of mental health professionals available in the area (Table 2).

**Table 2:** Model 1 - number of mental health professionals among DCI zip codes.

Mean number of mental health professionals					*F*	*p*-value
Distressed	At-risk	Mid-tier	Comfortable	Prosperous		
(*N* = 42)	(*N* = 20)	(*N* = 17)	(*N* = 10)	(*N* = 7)		
1.9	2.2	6.18	8.6	11.0	5.47	<0.001***

***Significant at the 0.001 alpha level.

Model 2 examined whether there was a significant difference in the level of DCI distress for particular zip codes and the number of mental health professionals available online for the area, while controlling for zip code population size. Results showed a significant difference between the levels of neighborhood distress and the number of mental health professionals available online in the zip code (*F* (4.91) = 2.91, *p* = 0.026). Distressed zip codes (*M* = 1.2 ± 4.6) were significantly different from prosperous zip codes (*M* = 5.6 ± 5.0) regarding the online availability of mental health service providers in the area (Table 3).

**Table 3:** Model 2 - number of mental health professionals online within particular DCI zip codes.

Mean number of mental health professionals					*F*	*p*-value
Distressed	At-risk	Mid-tier	Comfortable	Prosperous		
(*N* = 42)	(*N* = 20)	(*N* = 17)	(*N* = 10)	(*N* = 7)		
1.2	1.0	2.6	3.3	5.6	2.91	0.026*

*Significant at the 0.05 alpha level.

Models 3–6 examined the level of DCI distress for a particular zip code and the number of mental health provider specialties, while controlling for zip code population size. Results showed a significant difference between the level of DCI distress and the number of specialty mental health professionals who serve the area.

Model 3 examined significant differences in zip codes regarding LPCs. The number of LPCs significantly varied among the DCI zip codes (*F* (4.91) = 4.41, *p* = 0.003). Distressed zip codes (*M* = 0.81 ± 3.4) had significantly fewer LPCs than prosperous zip codes (*M* = 4.7 ± 4.5).

Model 4 examined significant differences in zip codes regarding practicing psychologists (PPsych). The number of PPsycs significantly varied among the DCI zip codes, (*F* (4.91) = 2.92, *p* = 0.024). However, the subsequent *post hoc* tests did not elucidate which means were different from one another (Table 4).

**Table 4:** Models 3–6 - types of mental health professionals.

Type of Mental Health Professional	Mean number of mental health professionals					*F*	*p-*value
	Distressed	At-risk	Mid-tier	Comfortable	Prosperous		
	(*N* = 42)	(*N* = 20)	(*N* = 17)	(*N* = 10)	(*N* = 7)		
Model 3. Licensed Professional Counselors (LPC)	0.81	1.1	3.2	4.1	54.7	4.41	0.003**
Model 4. Psychologists (Ppsych)	0.29	0.20	0.35	1.1	1.3	2.92	0.02*
Model 5. Social Workers (SW)	0.36	0.70	1.4	2.0	2.6	3.96	0.005**
Model 6. Marriage & Family Therapists (MFT)	0.07	0.10	0.60	0.70	0.43	3.68	0.008**

*Significant at the 0.05 alpha level.

**Significant at the 0.01 alpha level.

Model 5 examined significant differences in zip codes regarding SWs. The number of SWs significantly varied among the DCI zip codes, (*F* (4.91) = 3.96, *p* = 0.005). Distressed zip codes (*M* = 0.36 ± 1.7) had significantly fewer SWs than prosperous zip codes (*M* = 2.6 ± 1.7).

Model 6 examined significant differences in zip codes regarding MFTs. The number of MFTs significantly varied among the DCI zip codes, (*F* (4.91) = 4.68, *p* = 0.008). However, the subsequent *post hoc* tests did not elucidate which means were different from one another.

## Discussion

The present study served as an initial step in bringing awareness to what we have potentially identified as “mental health deserts.” Our results indicated preliminary evidence needing further examination toward our understanding of mental health deserts and their potential impact on mental health disparities. Based on the results, there was a significant relationship between the level of neighborhood distress and the availability of mental health professionals in the city of Houston. Specifically, distressed neighborhoods have significantly fewer mental health professionals compared to comfortable and prosperous neighborhoods. Similarly, when considering the availability of mental health professionals online, distressed neighborhoods have significantly fewer professionals compared to prosperous areas. The data showed a disproportionate availability of licensed mental health professionals, including licensed professional counselors, psychologists, social workers, and licensed family therapists. Distressed neighborhoods consistently showed lower numbers of available professionals compared to prosperous neighborhoods.

The present study utilized the conceptualization of the food desert model, applied to mental health, to the examination of geographical areas in need of increased mental health availability. Just as the consequences of food deserts are far more complex than once thought (24), likewise would mental health deserts be. Establishing mental health deserts as an area of mental health concern and the mechanisms that influence them will require more research and analysis. We began this examination of mental health deserts by assessing the availability of mental health professionals throughout socioeconomically diverse zip code areas in Houston, Texas. Our model of mental health deserts (Figure 2) proposes that the availability of mental health professionals may directly influence barriers and facilitators toward mental health service utilization. We present this data to initiate discourse and support an understanding of mental health deserts.

The impact of mental health deserts, and generally speaking, the lack of mental health care in disadvantaged communities, includes the well-known economic and personal health impacts such as missed work, negative affect, poor physical health, and poor quality of life, exacerbated by mental health comorbidities.

Notwithstanding, urban areas may face unique mental health challenges on a larger scale following natural disasters and other historical events. For instance, Houston, Texas faced natural disaster devastation from Hurricane Harvey in 2017, with mental health effects that are still being actualized today, such as grief, regret, and loss of possessions. Other psychological impacts were lasting anxiety, depression, and PTSD symptomology during large rainstorms, particularly during hurricane season (which entails June through November) (25). Of note, symptoms of PTSD, depression, and anxiety due to natural disasters are in line with literature on other natural disasters such as Hurricanes Katrina and Sandy (26). The COVID-19 pandemic has had widespread mental health impacts for adults and children alike. Financial and housing insecurity concerns remain. Disability and cognitive effects of Long.

COVID, which are the lingering effects of previous COVID-19 infection, directly contribute to mental health problems in communities (National Institutes of Health, 2025). These are concerns for all sectors of people. The pandemic affected millions globally. However, disadvantaged communities with limited resources and preexisting socially constructed concerns are most vulnerable in their ability to cope with uncontrollable disasters and seek treatment or engage in prevention (27, 28).

The findings also showed that online mental health professionals who operate within distressed and at-risk DCI communities were fewer than those in more advantaged areas. Although online mental health therapy is gaining popularity, individuals may, at some point, want in-person sessions. Nonetheless, the client-therapist relationship would be strengthened with in-person sessions that could be recommended or requested by either party. Therefore, mental health professionals who provide online mental health services are nonetheless still important for disadvantaged communities. Specific strategies to increase mental health service acquisition may include developing satellite clinics, mobile mental health units, and other innovative service delivery modes that enhance accessibility and address systemic barriers to care. Dissemination and collaboration with professional associations and academic institutions are also imperative for addressing the growing demand for mental health professionals in disadvantaged communities. Such partnerships can encourage new mental health professionals to deliver therapy and other mental health services (e.g., cognitive assessments) in these communities.

### Limitations of study

The present study is meant to initiate discourse on the identification of mental health deserts. While using a food desert model applied to mental health service consumption is conceptually innovative, there may be other models that are just as innovative and helpful. Taken alone, the present study’s results may oversimplify the complexity of mental health care accessibility because, as we proposed, mental health deserts are influenced by factors beyond proximity, such as cultural competence, stigma, affordability, insurance coverage, and other systematic factors. Additionally, this study took its sample of mental health providers from Psychology Today between the dates of August 1, 2023, through July 31, 2024. The availability and number of providers in areas are subject to change. Lastly, this study utilized the Distressed Community Index. While both databases are valuable resources, they may have limitations in accuracy and timeliness. Despite these limitations, the study was able to demonstrate a significant need for further investigation and likely targeted interventions to improve access to mental health care in underserved Houston areas.

### Implications and conclusion

We offer this study as a preliminary examination of *mental health deserts*. Although this study is preliminary and descriptive, it has underscored the shortage of mental health professionals in economically distressed communities and highlighted the critical need for targeted efforts to address mental health disparities socio- geographically. The findings provided insights into mental health and service acquisition in the city of Houston and similar socio-geographic cities. This examination of mental health deserts has implications for mental health research, professionals, and stakeholders in the mental health field. Mental health practitioners may leverage these findings to advocate for increased funding and significant policy reforms aimed at reducing disparities in mental health care access. Efforts may include advocating for comprehensive insurance coverage for mental health services and other healthcare system policies, and advocating for greater investment in community-based mental health infrastructure. Additionally, community-based engagement is needed to understand exactly what mental health services would be most beneficial to communities, keeping in mind their culture and daily lives. The example model presented in Figure 2 will require significant additional analyses and a greater depth of data collection to test, confirm, and fully conceptualize, which we will pursue through future research. We understand this and intend for the present study to provide other interested researchers a springboard to build upon such examination.

Addressing mental health resource shortages requires robust strategies tailored to the needs of distressed communities at different levels of implementation. More research is needed regarding the present study’s findings and the conceptual model of mental health deserts set forth. Researchers and mental health professionals are needed to disseminate mental health resources to community members and policy stakeholders with the intent to ensure equitable access to mental health care across diverse socioeconomic neighborhoods and particularly for disadvantaged communities.

## Data Availability

The data used in this study was curated from publicly available datasets. The curated dataset used in this study is available upon request from the corresponding author. Data was accessed from Economic Innovation Group, Distressed Community Index (DCI) at https://eig.org/dci-hub/ on November 11, 2024 and from Psychology Today’s Find a Therapist directory at https://www.psychologytoday.com/us/therapists/tx/houston on November 12, 2024.
